# Altered spring phenology of North American freshwater turtles and the importance of representative populations

**DOI:** 10.1002/ece3.4120

**Published:** 2018-05-04

**Authors:** Fredric J. Janzen, Luke A. Hoekstra, Ronald J. Brooks, David M. Carroll, J. Whitfield Gibbons, Judith L. Greene, John B. Iverson, Jacqueline D. Litzgus, Edwin D. Michael, Steven G. Parren, Willem M. Roosenburg, Gabriel F. Strain, John K. Tucker, Gordon R. Ultsch

**Affiliations:** ^1^ Department of Ecology, Evolution & Organismal Biology Iowa State University Ames Iowa; ^2^ Department of Integrative Biology University of Guelph Guelph ON Canada; ^3^ Warner New Hampshire; ^4^ Savannah River Ecology Laboratory Aiken South Carolina; ^5^ Department of Biology Earlham College Richmond Indiana; ^6^ Department of Biology Laurentian University Sudbury ON Canada; ^7^ Division of Forestry and Natural Resources West Virginia University Morgantown West Virginia; ^8^ Hinesburg Vermont; ^9^ Department of Biological Sciences Ohio University Athens Ohio; ^10^ GAI Consultants, Inc. Bridgeport West Virginia; ^11^ Jerry F. Costello National Great Rivers Research and Education Center Confluence Field Station East Alton Illinois; ^12^ Department of Biology University of Florida Gainesville Florida

**Keywords:** advancing phenology, climate, nesting, phenotypic plasticity, representative population, reptile

## Abstract

Globally, populations of diverse taxa have altered phenology in response to climate change. However, most research has focused on a single population of a given taxon, which may be unrepresentative for comparative analyses, and few long‐term studies of phenology in ectothermic amniotes have been published. We test for climate‐altered phenology using long‐term studies (10–36 years) of nesting behavior in 14 populations representing six genera of freshwater turtles (*Chelydra*,* Chrysemys*,* Kinosternon*,* Malaclemys*,* Sternotherus*, and *Trachemys*). Nesting season initiation occurs earlier in more recent years, with 11 of the populations advancing phenology. The onset of nesting for nearly all populations correlated well with temperatures during the month preceding nesting. Still, certain populations of some species have not advanced phenology as might be expected from global patterns of climate change. This collection of findings suggests a proximate link between local climate and reproduction that is potentially caused by variation in spring emergence from hibernation, ability to process food, and thermoregulatory opportunities prior to nesting. However, even though all species had populations with at least some evidence of phenological advancement, geographic variation in phenology within and among turtle species underscores the critical importance of representative data for accurate comprehensive assessments of the biotic impacts of climate change.

## 1 INTRODUCTION

Global climate has warmed substantially and at an accelerating rate in recent decades (IPCC, 2014), although some regions have warmed more slowly (Pan et al., 2004). Diverse biotas are responding to this climatic change in various ways (Bell et al., 2015; Gibbs & Breisch, 2001; Li, Cohen, & Rohr, 2013; Parmesan & Yohe, 2003; Root et al., 2003; Thackeray, Jones, & Maberly, 2008). Emerging from large‐scale analyses of longitudinal field studies of these phenomena is the conclusion that altered phenology (i.e., timing of life‐cycle events) is a key biotic response to climate change. Populations of numerous taxa, from birds to butterflies to angiosperms, are advancing the annual onset of fundamental biological activities, occasionally with documented effects on fitness (Benard, 2015; Pike, Antworth, & Stiner, 2006).

Many reports of phenological shifts, however, document the response of single populations often near the edge of a species’ range. Summaries of these individual studies typically assume that conspecific populations will respond similarly to climate change and, therefore, use a single datapoint per species (Brown et al., 2016; Parmesan, 2007; Parmesan & Yohe, 2003). This practice obscures intraspecific variation in phenological responses to climate change and potentially inhibits mechanistic understanding of phenological shifts that population comparisons afford. Boundary populations may differ greatly from conspecific populations toward the center of the geographic range (Angert & Schemske, 2005). One reason is that boundary populations are more likely to be limited by abiotic factors than are more central populations. For example, in the northern temperate zone, populations at the northern edge of their species’ range are more thermally limited than are conspecific populations farther from the range boundary (Gilman, Wethey, & Helmuth, 2006; Root, 1988). Niche modeling of 108 reptile species endemic to the United States supports the idea that climatic factors are the primary cause of poleward range limits, whereas southern ranges of these species are more likely limited by nonclimatic factors (Cunningham, Rissler, Buckley, & Urban, 2015). Because climate warming is occurring more rapidly toward the polar regions (IPCC, 2014; Karl & Trenberth, 2003), populations closer to the poles may exhibit more substantive phenotypic responses than conspecific populations located toward the center of the range (Mazaris, Kallimanis, Pantis, & Hays, 2013; Rosenblatt, Crowley, & Schmitz, 2016) and, hence, neither are necessarily representative of the entire species. Evolutionarily, however, marginal populations may be the least suited to respond to steepening environmental gradients because of genetic drift as well as gene flow from populations in other environments (Peischl, Kirkpatrick, & Excoffier, 2015; Polechová & Barton, 2015). All these factors challenge the assumption that conspecific populations will respond similarly to climate change and thus can be represented by a point estimate.

Reviews of biotic responses to climate change have incorporated a wealth of data from a variety of species, but the data sets still contain notable taxonomic gaps. In particular, few studies of long‐term phenology of ectothermic amniotes (=nonavian reptiles) have been available for comparison (Table S1). Although such studies are beginning to appear in the literature (Urban, Richardson, & Freidenfelds, 2014), this paucity nonetheless may reflect the noteworthy challenges in accurately observing life‐history events in these often‐secretive taxa over many years (Frazer, Greene, & Gibbons, 1993). Moreover, this group exhibits numerous biological features linked strongly to temperature (e.g., many have temperature‐dependent sex determination (Bull, 1980; Janzen & Paukstis, 1991) and a number of species are already imperiled (Turtle Taxonomy Working Group, 2017; Ihlow et al., 2012)), thus illuminating both the scientific importance and practical urgency of the issue.

We combine long‐term field data on nesting behavior in 14 populations representing six genera of North American freshwater turtles, along with spring emergence data from three populations representing three genera, to investigate effects of accelerating climate change on phenology. Because of the biological significance of nesting behavior and for ease of comparison among independent field studies, we focused on date of the first nesting event in a population in a given year as a measure of phenology. We used these data first (i) to document annual variation in nesting phenology and identify populations and species with advancing nesting phenology (i.e., initiating the nesting season earlier in more recent years). We then (ii) assessed the extent to which geography contributed to the observed patterns, with special focus on assessing the biophysical and climatological prediction that populations at the northern boundary of a species’ range in the northern hemisphere should exhibit the most significant temporal responses. In this context, we also (iii) explored local climatic thermal cues that might be mechanistically related to annual variation in nesting phenology. To evaluate mechanisms (phenotypic plasticity vs. genetic adaptation) that underpin within‐population patterns of annual variation in nesting phenology, we (iv) interpret our findings in light of available population‐level data for annual variation in key prenesting activities (i.e., phenological traits related to spring emergence from hibernation) and individual‐level data for annual variation in onset of nesting (e.g., is earlier nesting in more recent years driven by older females [within‐generation ~ plasticity] or by primiparous females [across‐generations ~ adaptation]?).

## 2 METHODS

### 2.1 Data collection

We focused on six genera from three families of North American freshwater turtles whose reproductive biology has been studied intensively in multiple populations from Nebraska, Illinois, South Carolina, Maryland, and Ontario over at least a 10‐year period (Table 1).

**Table 1:** List of species, locations, years sampled, and phenological trait(s) reported

Species	Locality	Latitude, longitude	Years (*N*)^a^	Trait
*Chelydra serpentina*	Algonquin Provincial Park, ON	45.54N, 78.27W	1976–2011 (36)	First nest
*Chelydra serpentina*	Thomson Causeway Recreation Area, IL	41.95N, 90.11W	1989–2012 (23)	First nest
*Chelydra serpentina*	Crescent Lake National Wildlife Refuge, NE	41.73N, 102.3W	1981–2013 (23)	First nest
*Chelydra serpentina*	Sand Run Lake, WV	39.07N, 79.38W	1988–2006 (18)	First emergence
*Chelydra serpentina*	Sand Run Lake, WV	39.07N, 79.38W	1988–2007 (19)	First hibernation^b^
*Chelydra serpentina*	Savannah River Site, SC	33.34N, 81.74W	1977–1998 (9)	First nest
*Chrysemys picta*	Algonquin Provincial Park, ON	45.54N, 78.27W	1985–2011 (26)	First nest
*Chrysemys picta*	Thomson Causeway Recreation Area, IL	41.95N, 90.11W	1989–2013 (25)	First nest
*Chrysemys picta*	Crescent Lake National Wildlife Refuge, NE	41.73N, 102.3W	1986–2013 (20)	First nest
*Chrysemys picta*	Two Rivers National Wildlife Refuge, IL	38.99N, 90.55W	1995–2010 (15)	First nest
*Clemmys guttata*	Warner, NH	43.29N, 71.83W	1988–2012 (25)	First emergence
*Glyptemys insculpta*	Monkton, VT	44.27N, 73.12W	1986–2012 (19)	First basking
*Kinosternon flavescens*	Crescent Lake National Wildlife Refuge, NE	41.73N, 102.3W	1982–2013 (17)	First nest
*Kinosternon subrubrum*	Savannah River Site, SC	33.34N, 81.74W	1977–2003 (10)	First nest
*Malaclemys terrapin*	Patuxent River, MD	38.50N, 76.70W	1987–2005 (18)	First gravid^c^
*Malaclemys terrapin*	Poplar Island, MD	38.76N, 76.38W	2004–2013 (10)	First nest^c^
*Sternotherus odoratus*	Two Rivers National Wildlife Refuge, IL	38.99N, 90.55W	1995–2011 (13)	First nest
*Trachemys scripta*	Two Rivers National Wildlife Refuge, IL	38.99N, 90.55W	1994–2012 (19)	First nest
*Trachemys scripta*	Savannah River Site, SC	33.34N, 81.74W	1977–2003 (16)	First nest

a Range of years sampled with total number of years sampled in parentheses. Note that some studies were not contiguous.

b First hibernation is the date the first turtle was observed to enter hibernation.

c These data were combined for analyses. See 2 for justification.

We collected long‐term nesting data on one population of *Kinosternon flavescens*, one population of *K. subrubrum*, four populations of *Chelydra serpentina*, four populations of *Chrysemys picta*, one population of *Sternotherus odoratus*, two neighboring populations of *Malaclemys terrapin*, and two populations of *Trachemys scripta* (Table 1, Figure S1). The primary nesting phenology data set encompassed 280 monitor‐years at six research sites between 1976 and 2013, with individual efforts encompassing periods of field study from 10 to 36 years (mean = 24; Table 1).

At each of the six field sites, three of which were near the northern edge of the range for the genera *Kinosternon, Chelydra*,* Chrysemys*, and *Trachemys* (see Turtle Taxonomy Working Group, 2017 for species’ range maps), experienced personnel monitored the areas prior to onset of the nesting season (Carroll & Ultsch, 2007; Gibbons, 1990; Iverson, 1991; Iverson & Smith, 1993; Pfau & Roosenburg, 2010; Riley & Litzgus, 2013; Schwanz & Janzen, 2008; Schwarzkopf & Brooks, 1985; Strain, Anderson, Michael, & Turk, 2012; Tucker, Dolan, Lamer, & Dustman, 2008). Onset was indicated when the first gravid turtle was observed nesting, which we recorded as day of the year for statistical analyses. From 1995 to 2005, the first nesting date for the *Malaclemys* population from Patuxent, Maryland was not available, so first gravid date, as determined by palping the inguinal area for shelled eggs, was used instead. For these years, we estimated first nesting date from the relationship between first gravid date and first nesting date previously established for this population between 1987 and 1994. We focused on first nesting date because it is widely available for the populations studied and we hypothesized it would respond in a direct, linear way to climate change. Whereas first nesting date often may be significantly correlated with median (or mean) nesting date (Tucker et al., 2008), median nesting date can obscure changes in the underlying population dynamics of multivoltine species (Schwanz & Janzen, 2008). Furthermore, we note that first nesting date and the first major pulse of nesting activity are highly correlated (e.g., *R* ^*2*^ = .92 for our Illinois *Trachemys* population). To further clarify relationships between spring climate and phenology in North American freshwater turtles, we also examined data from long‐term studies of spring emergence from hibernation of *Chelydra* in West Virginia and *Clemmys guttata* in New Hampshire and of onset of spring thermoregulatory (i.e., aerial basking) behavior of *Glyptemys insculpta* in Vermont. These three studies were of similar duration to our nesting studies (mean = 24 years; Table 1).

We obtained air temperature data from weather stations within 1–30 km of each field site from the National Climatic Data Center (ncdc.noaa.gov) for the USA and from Environment Canada (climate.weather.gc.ca) for Canada. We calculated heating degree‐days (HDD) as the sum of the number of degrees Fahrenheit that each daily mean temperature fell below 65°F (~18°C; Strachey 1878) for 1–28 February, 1–31 March, 1–30 April, and 1–31 August. The base temperature (i.e., 65°F) represents a minimum thermal threshold below which freshwater turtles cannot perform many tasks necessary for energy acquisition and allocation (Bulte & Blouin‐Demers, 2010; Edwards & Blouin‐Demers, 2007). Note that higher HDD values indicate cooler temperatures. Such degree‐day models can provide useful mechanistic explanations of phenological change (Bell et al., 2015; Cayton, Haddad, Gross, Diamond, & Ries, 2015; Williams, Stichter, Hitchcock, Polgar, & Primack, 2014). As employed here, this climate metric integrates thermal variation prior to onset of the reproductive season (here, starting in late April–June), emphasizing spring conditions that could impact onset of the nesting season due to temporal proximity (Iverson, Higgins, Abby, & Griffiths, 1997). Relationships between first nesting date and HDD for April were similar to those between first nesting date and mean April temperature (Table S8).

### 2.2 Statistical approach and model selection

Testing for temporal trends in phenology and links to climate primarily involved estimating the relationship (i.e., the slope) between the discrete timing of phenological events and a continuous predictor (i.e., year or climatic factor). We determined the optimal random and fixed components of our statistical models using the top‐down approach (described in Zuur, Ieno, Walker, Saveliev, & Smith, 2009) and the sample‐size‐corrected Akaike information criteria (AICc). Because we wanted to estimate potential temporal and climatic effects on phenology for each species and population, and because the populations sampled were unlikely to represent random samples of their species distributions, when justified we fit population and species as fixed effects. For all analyses, when estimating rates of change for multiple sites (i.e., fitting a common slope), we also compared our reported estimates (Tables S2–S6; Table 2) to those from varying intercept mixed models with site fit as a random effect. These estimates were always well within error of each other. Due to potential interactions between year and species, we then used ANCOVA to test for heterogeneity of slopes. When possible, we fit a common slope to estimate the rate of change at the highest justifiable grouping of populations. When we could not fit a common slope for all populations, we split populations by species. When we could not fit a common slope to all populations within a species, we estimated separate slopes for each population. In particular, we combined data on *Malaclemys* populations from Patuxent, Maryland, and Poplar Island, Maryland after ANCOVA tests failed to find a significant effect of site (i.e., the populations have responded similarly to temporal and climatic variation). There was minimal autocorrelation in our time series (Durbin–Watson test, *p > *.2 for all populations), thus we considered linear regression analyses appropriate. We inspected all data and residuals for assumptions of normality and conducted all tests in R version 3.1.2 (R Core Team 2015), employing a two‐tailed alpha of 0.05 (except where noted).

**Table 2:** Estimates of the phenological response to climatic variation from linear regressions of first nesting date on heating degree‐days (HDD) for April. Rate of change reflects an estimate from the regression slope. “All populations” represents a regression using data from all 14 populations, with the common slope estimate justified by a comparison of slopes test (black line, Figure 3a). Separate regressions were used to independently estimate change in nesting date for each species and population. Bold text indicates significance at α = 0.05 level

Species‐site	Rate of change (days per 100 degree‐days)	*SE*	*N*	*F*	*p* c	*R* b
All populations^a^	4	0.5	280	62.3	**<.001**	.75
*Chelydra serpentina* a	3.4	0.7	91	59.5	**<.001**	.72
Algonquin Provincial Park, ON	3.4	1.1	36	10	**.002**	.2
Crescent Lake National Wildlife Refuge, NE	2.7	1	23	6.83	**.008**	.21
Thomson Causeway Recreation Area, IL	4.9	1.5	23	11	**.002**	.31
Savannah River Site, SC	0.9	5.9	9	0.02	.444	0
*Chrysemys picta* a	4.1	0.9	86	18.1	**<.001**	.45
Algonquin Provincial Park, ON	4.3	1.3	26	10.4	**.002**	.27
Crescent Lake National Wildlife Refuge, NE	2.5	2.5	20	1.02	.163	.33
Thomson Causeway Recreation Area, IL	4.1	1.5	25	7.69	**.005**	.22
Two Rivers National Wildlife Refuge, IL	5.4	2.4	15	5	**.022**	.22
*Trachemys scripta* a	6.2	2.3	35	13.5	**.006**	.42
Two Rivers National Wildlife Refuge, IL	7	2.1	19	11.4	**.002**	.37
Savannah River Site, SC	2.2	6.8	16	0.1	.376	0
*Kinosternon* spp.^a^	3.3	1.9	27	69.9	**.048**	.84
Crescent Lake National Wildlife Refuge, NE	2	1.2	17	2.67	.062	.09
Savannah River Site, SC	13.2	7.6	10	3.02	.06	.18
*Sternotherus odoratus*						
Two Rivers National Wildlife Refuge, IL	4.1	3.1	13	1.76	.106	.06
*Malaclemys terrapin*						
Poplar Island, MD^b^	5	1.9	28	7.24	**.006**	.16

a Population was included as an independent variable in these models, significantly improving the statistical fit.

b This includes data from Patuxent, MD, and Poplar Island, MD.

c Significance calculated from a one‐tailed *t* test for a positive slope.

### 2.3 Testing for temporal change in phenology

To evaluate consistency in temporal changes in phenology, we regressed date of first nesting (or other phenological measure) against year. In addition to our attempts to identify congruence in the response to climate change using ANCOVA, to aid comparison between temporal and climatic variation in phenology, we fit separate regressions for each species and population (Tables 2 and S2, Tables S4 and S5). This means that some slope estimates made at the species level or higher, as noted in Table S2, were provided for illustrative purposes, despite evidence of significant heterogeneity among populations comprising these groupings.

### 2.4 Assessing the explanatory power of geography

To assess whether temporal patterns in nesting phenology might be related to geography, we compared regression slope estimates of the relationship between first nesting date and year. For species with distinct populations, we plotted estimates of phenological advancement by latitude (Figure S2). We also calculated the Pearson's product moment correlation between rate of advancement and latitude for each species and performed a one‐tailed test for the significance of this correlation based on the hypothesis that change in the onset of nesting would be greater at higher latitudes (i.e., higher latitudes would have a more negative slope).

### 2.5 Identifying potential climatic factors affecting phenology

To explore climatic variation that might be mechanistically related to annual variation in nesting phenology, we adopted a similar statistical approach as above. We modeled the onset of nesting season using measures of HDD summarizing climatic variation during the preceding months. Model comparison using HDD for February, HDD for March, HDD for April and all covariate combinations showed that models containing solely HDD for April were favored by AICc. For all populations, we also evaluated possible correlation or covariation with climate indices (“winter” and monthly means of the Northern Atlantic Oscillation index (NAO), monthly means of the Pacific North American index (PNA), monthly means of the Southern Oscillation Index (SOI), and 3‐months averages of the Oceanic Niño Index (ONI)), all downloaded from the NOAA Climate Prediction Center (cpc.ncep.noaa.gov; Table S6). Again, model selection favored models containing only HDD for April. Once we determined the optimal covariate structure, we again employed ANCOVA and linear regression to estimate relationships between the onset of nesting and HDD for April.

### 2.6 Testing the relationship between prenesting and nesting behavior

To interpret our nesting phenology findings in light of key prenesting activities, we applied the same model selection and regression approach to evaluate temporal and climatic trends for first emergence from winter hibernation and for initial observation of spring basking. Model selection favored models containing only HDD for February to explain variation in the onset of spring emergence and basking, and similarly, only HDD for August to explain variation in the onset of hibernation.

### 2.7 Examining the evidence for contemporary climate change

Lastly, we assessed temporal trends in HDD (i.e., climate change) using a similar combination of ANCOVA and linear regression, except that we also estimated the rate of change in HDD for a subset of sites containing at least one population with evidence of advancing nesting phenology. We evaluated the sensitivity of this estimate to unequal sampling across sites by subsampling the *X*‐axis for years where at least 2 (of 6), at least 3 (of 6), at least 4 (of 6), or at least 5 (of 6) sites were represented. The reported regression using the full range of data provided a relatively minimal estimate of the rate of warming (range of slope estimates = −16.2 to −40.4 HDDs for April per decade). Of note, the greatest rate of spring warming was estimated from recent years (1994–2011) for which five (of six) sites were represented (−40.4 HDDs for April per decade, *R* ^2^ = .89). For species with distinct populations, we also plotted our estimates of phenological advancement by the rate of change in HDD for April (Figure S2b). We then calculated the Pearson's product moment correlation between the rate of advancement in phenology and the rate of decline in HDD for April (i.e., the rate of spring warming) for each population and performed a one‐tailed test for the significance of this correlation based on the hypothesis that the rate of advancement would be greater for populations that have experienced a greater decline in HDD (i.e., more warming).

## 3 RESULTS

### 3.1 Temporal trends

**Figure 1:**
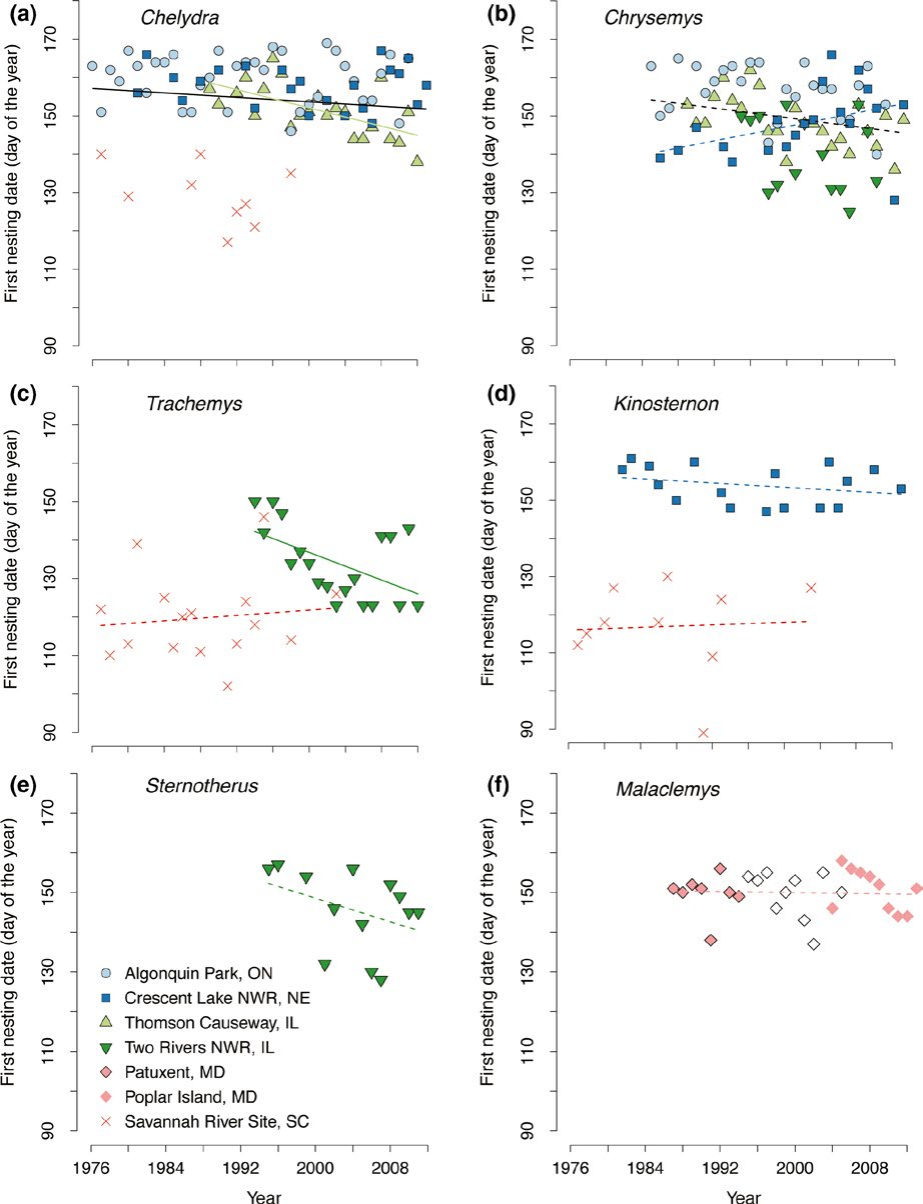
The first nesting date of freshwater turtles has advanced in the past 36 years for most populations studied in the northern United States and Canada, although the magnitude and significance of this advancement have varied among species and populations. Different symbols and colors represent different populations. Solid lines indicate linear regressions with significant, negative slopes (*p *<* *.05). Dashed lines represent linear regressions with slopes not significantly different from zero (*p *>* *.05). Black lines are from regressions of multiple populations grouped at the species level (see Table S2). Colored lines are regressions from single populations, typically highlighting populations that differed significantly in their phenological response relative to other populations of the species. (a) The solid black line was estimated from all four populations of *Chelydra serpentina*, but the solid green regression line for Thomson Causeway, IL illustrates significant variation in the magnitude of phenological advancement among these populations. (b) *Chrysemys picta* from Crescent Lake National Wildlife Refuge, NE (dashed blue line) have a significantly different slope from the other three populations, preventing precise estimation of this species rate of phenological change. (c) The nesting phenology of a northern *Trachemys scripta* population has significantly advanced, while a more southern population has not. (d) *Kinosternon flavescens* from Crescent Lake National Wildlife Refuge, NE (dashed blue line) and *K. subrubrum* from the Savannah River Site, SC (dashed red line) show possible latitudinal differences in the advancement of nesting phenology, but these differences could also represent species‐specific responses. (e) The single population of *Sternotherus* studied shows a nonsignificant temporal trend in nesting phenology. (f) The nesting phenology of *Malaclemys* populations has been relatively static across the time period studied. Note here the open symbols represent estimated first nest dates calculated from first gravid dates based on the relationship between first nest date and first gravid date established at this site

All populations exhibited annual variation in date of first nesting. Eleven of the 14 populations examined displayed negative trends with respect to time (Table S2; Figure 1), which is more than expected by chance (one‐sided sign test, *p *=* *.03, Cohen's *h* = 0.59), but only three of these comparisons were individually significant (i.e., *p *<* *.05 without adjusting for multiple comparisons). Still, 79% of the examined populations began the nesting season earlier than they did at the beginning of the respective field studies. The advance in onset of the nesting season for populations from the initial year of fieldwork to the last year of study varied from as few as 0 day to as many as 27 days (Table S2; Figure 1). Perhaps most notably, by 2012, the Illinois population of *Trachemys* initiated the nesting season over 3 week earlier than it did in the mid‐1990s (from 30 May 1994 to 3 May 2012).

**Figure 2:**
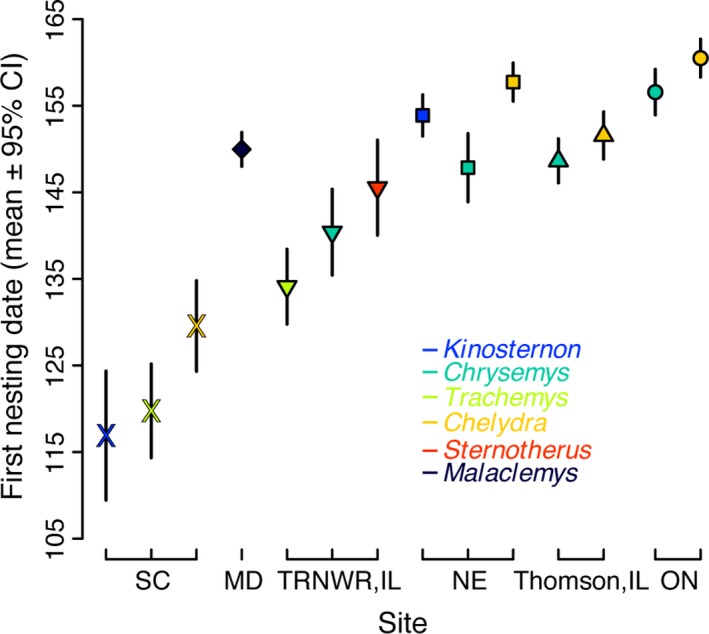
Mean first nesting date (±95% CI) for 14 populations of freshwater turtles showing the relative contribution of site (different shapes) and species (different colors). Sites are presented in ascending order by latitude

Onset of the nesting season also varied among years for each species (Figure 1), and mean first nesting date varied among species (Figure 2). All species studied except *K. subrubrum* tended to nest earlier through time, with populations from three of seven species doing so significantly earlier (Table S2; Figure 1) and another one nearly so (*S. odoratus*,* p *<* *.10). Where it occurred, *Chelydra* was the last species to initiate the nesting season in a given year compared to the other species studied at a given location. That is, where comparisons can be made, the smaller turtle species (*Sternotherus* and *Kinosternon*) tended to nest earlier at a particular site than the moderately sized species (*Chrysemys* and *Trachemys*), which in turn began nesting sooner than the larger‐bodied *Chelydra* (Figure 2).

### 3.2 Geographic trends

Geography exerted a noticeable effect on both mean first nesting date (Figure 2) and phenological advancement of nesting (Figure 1), but these effects were inconsistent with expectations. Focusing on species with at least two distinct populations, as described above, the northern range‐edge population of *Trachemys* in Illinois (Figure 1c) exhibited the most striking advancement in the onset of nesting among all populations studied (−9.0 days/decade; Table S2). By comparison, the *Trachemys* population in South Carolina, from a more central position in the geographic range of this species, exhibited no evidence of advancement in the onset of nesting date (+1.7 days/decade; Table S2). Limiting the comparison of *Trachemys* populations to years with overlapping samples (1994–2003) did not qualitatively change these slope estimates. This geographic pattern was essentially reversed for northern range‐edge vs. range‐center populations of *Chelydra* and *Chrysemys*. Ontario populations of both species only modestly advanced the onset of the nesting season in more recent years compared to the northern Illinois populations of these species that are closer to the latitudinal centers of their respective geographic ranges. The southernmost populations studied of these species (South Carolina and southern Illinois, respectively) advanced their nesting phenology at similar rates (Table S2). Nebraska populations of *Chelydra* and *Chrysemys* showed the least evidence of phenological advancement for each species, with the *Chrysemys* population actually trending toward later nesting, further complicating a simple interpretation of the influence of geography (i.e., latitude). Even so, we did not detect anomalous trends in the climatic factors identified to be important for nesting onset at the Nebraska site that could explain this inconsistency (Table S3). Taken together, we found no consistent latitudinal pattern in temporal changes in the onset of nesting within species (Figure S2) and no significant correlation between latitude and the magnitude of phenological change in nesting among species (*r*_8_ = .07, *p *=* *.58).

### 3.3 Climatic cues

**Figure 3:**
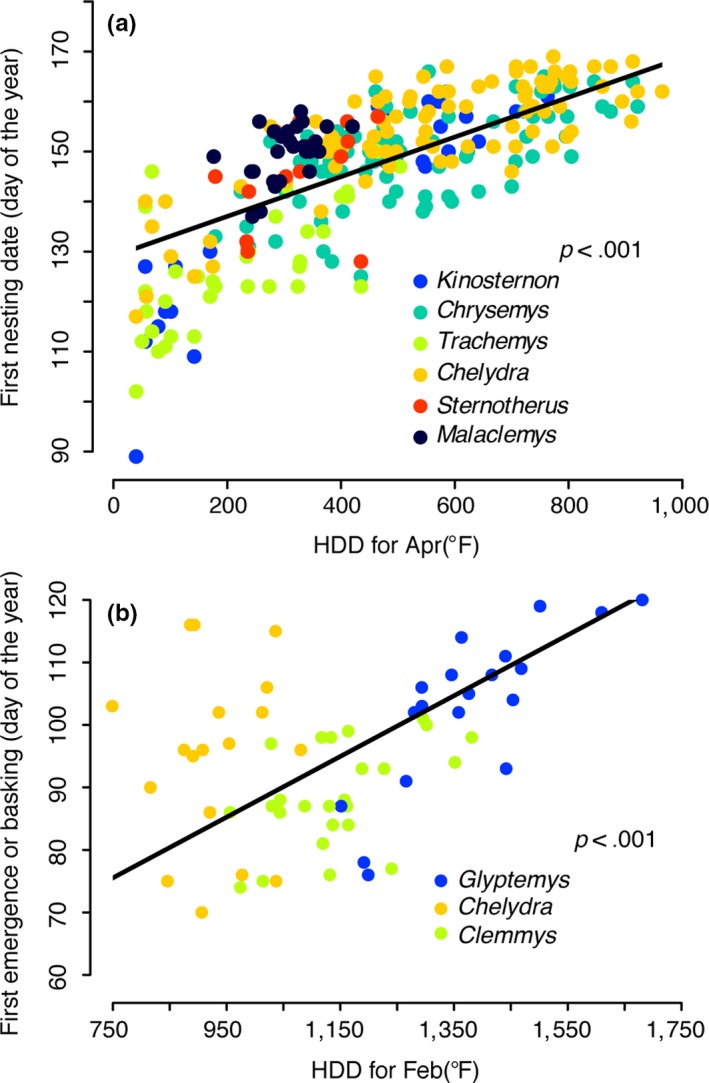
Spring phenologies of freshwater turtles are positively associated with a single climatic factor. (a) First nesting date is positively associated with heating degree‐days (HDD) for April (*p *<* *.001). Different colors represent different species as in Figure S1. The solid black line represents a common regression slope for all 14 populations studied from a best‐fit model that included population as an additive effect. There was no significant effect of population on slope of the regression line (Population × Year, *p *=* *.66). There was significant heterogeneity in the slope of the regression line among species (Year × Species, *p *<* *.05), however, all species‐specific slope estimates were positive and all except *Sternotherus* were significantly so. Table 2 enumerates variation in this relationship within and among species. (b) Spring emergence of freshwater turtles is also positively associated with a single climatic factor, heating degree‐days (HDD) for February, which summarizes thermal variation immediately preceding spring emergence. The solid black line represents a common regression slope for three populations with estimates of spring emergence, justified by a comparison of slopes test (ANCOVA: Year × Population, *p *>* *.05). Separate regression estimates for each population are listed in Table S5

Nesting phenology was strongly linked to spring temperature, as summarized by heating degree‐days for April (HDD for April; Figure 3a). Nearly all populations nested early when April was warmer, 8 of 14 populations significantly so (Table 2). HDD for April also significantly changed with time when all field sites were considered together (Table S3 “All sites”). Larger‐scale climate indices such as the NAO, PNA, SOI, and ONI did not explain substantial variation in nesting date and including these indices as covariates did not improve our ability to predict nesting date (Table S6).

Focusing on the Illinois populations of *Trachemys* (northern edge of the species’ geographic range) and *Chrysemys* (north‐central portion of the species’ geographic range but farther north than the *Trachemys* population) illustrates the general relationship between spring temperature and nesting phenology. For these two populations, HDD for April varied inversely with time (*r *=* *−.44, *p *=* *.060 and *r *=* *−.30, *p *=* *.151, respectively) and positively with date of first nesting (*r* = +.63, *p *=* *.004 and *r* = +.50, *p = *.011, respectively). In other words, annual April climate warmed and this warming coincided with an earlier onset of the nesting season in both populations. In fact, the southern Illinois site was the locality with the greatest evidence of climate warming (Table S3) and its *Trachemys* population showed the greatest advancement in nesting phenology (Table S2, Figure 1c). Furthermore, sites with little to no evidence of progressively warmer springs (South Carolina and Poplar Island, Maryland) harbored populations of freshwater turtles with no evidence of progressively earlier nesting, despite these populations having correspondingly strong relationships between nesting onset and HDD for April (Tables S2 and S3; Table 2). For the same set of populations, we used to test the influence of latitude on the rate of phenological advancement, the rate of change in HDD for April better predicts temporal change in nesting phenology (Figure S2; *r*_8_ = .50, *p *=* *.07).

### 3.4 Prenesting activities

**Figure 4:**
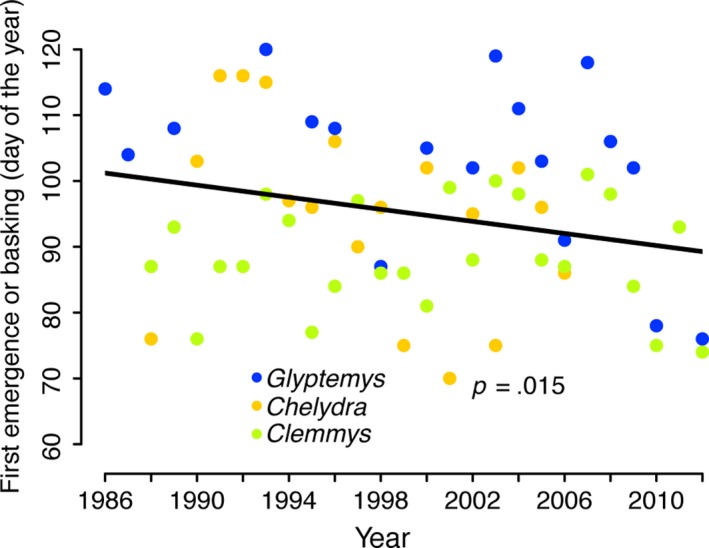
First spring emergence or first basking of freshwater turtles has advanced significantly in the past 25 years. The solid black line represents a common regression slope for three populations with estimates of spring emergence. A comparison of slopes test justified fitting a common slope (ANCOVA: Year × Population, *p *>* *.05). Separate regression estimates for each population are listed in Table S4

The phenological patterns of two additional traits (first day of spring emergence from hibernation and first day basking) for three separate populations and species exhibited temporal trends (Table S4, Figure 4) that mirrored those we described above for onset of the nesting season. Spring emergence and basking patterns were also similar to those detected for nesting activities with respect to taxonomic and geographic variation. Furthermore, these two traits similarly covaried with spring temperature (Table S5; Figure 3b). Spring emergence behavior did not depend on what date the turtles entered hibernation (at least for *Chelydra* in West Virginia), but rather on how quickly spring warmed. In other words, despite variation among taxa and localities, multiple thermally linked phenological traits of freshwater turtle populations in North America commonly began sooner in more recent years.

## 4 DISCUSSION

Our long‐term field studies of freshwater turtle populations in North America occurred over a period of increasingly rising global temperatures (IPCC, 2014). Our assessment is among the first to provide long‐term data on intraspecific and interspecific patterns of phenology for ectothermic amniotes. Although implying linkage between changing climate and critical behaviors, the results of our study are not wholly consistent with predictions that populations at a range edge will respond to climate change differently than populations in the center of a species’ range, highlighting prominent intraspecific variation.

Despite overall consistency in responses of nesting behavior to spring temperature, not all turtle populations responded to warmer springs to the same degree or, in one case, the same direction. Variation in the onset of nesting could derive from multiple sources. Life‐history variation, and variation in the underlying physiology, could have influenced responses of nesting behavior to climate conditions. For example, egg follicles develop in the fall in *Chelydra*, but develop in both fall and spring in *Chrysemys* (Rollinson, Farmer, & Brooks, 2012), thereby potentially contributing to both within‐locality annual variation among species and among‐locality annual variation within species in the onset of nesting season because nesting cannot commence until follicles are fully developed and then shelled (Ewert, 1979). Additional variation in phenology could be driven by plastic responses to other environmental factors, including water temperature, cloudiness, and precipitation events (Bowen, Spencer, & Janzen, 2005), although note that we did not find a link between hibernation entry and hibernation departure for the one population with available data. This interpretation of predominately plastic phenological responses to local, temporally proximate conditions (vs. genetic adaptation) is supported by other research at our field sites. Specifically, capture‐mark‐recapture studies in these populations without exception identify different marked individuals as initiating the nesting season each year as opposed to new, unmarked females (Schwanz & Janzen, 2008). Thus, at least over the time frame of our field studies, plasticity appears to be the primary mechanism underlying the observed phenological patterns below the species level, consistent with interpretations of most studies of responses to climate change (Urban et al., 2014).

One important conclusion of this comparative study is that inadequate geographic sampling could skew assessments of the biotic impacts of climate change. Populations at higher latitudes within a species’ range may be more likely to experience climate change (IPCC, 2014) and could potentially be more sensitive to those thermal changes (Cunningham et al., 2015). Illustrating this issue, the Illinois populations of *Trachemys* at the northern edge of its species’ range exhibited a stronger phenological response to climate change than the more northern Illinois population of *Chrysemys* that is more central to its species’ range. This pattern of response is explained by the greater degree of warming experienced at the more southern Illinois site, but not predicted by simple latitudinal trends in climate change prediction models. Nevertheless, disproportionate representation of populations near range limits (either poleward or equatorward) in a data set could lead one to overestimate the strength of response of a species to climate change. Moreover, the velocity of climate warming through 2100 is generally predicted by large‐scale global climate models to be higher in continental interiors relative to localities closer to coasts (Loarie et al., 2009), whereas regionally downscaled climate models do not always concur (Pan et al., 2004). Thus, the choice of representative populations can affect both pattern and projection.

We focused our analyses on date of the first observed behavior to assess phenological variation. This emphasis promoted ease of comparison among our independent research programs and is consistent with most literature on phenological responses to climate change. Indeed, various shorter‐term studies of freshwater turtles had already suggested that onset of nesting season might be linked to proximate thermal conditions (Congdon, Breitenbach, Sels, & Tinkle, 1987; Iverson et al., 1997). Interestingly, however, most work on marine turtles has noted thermally linked temporal changes in median nesting date, but not in onset of the nesting season (Table S1). We therefore recognize that this trait might not reflect population response to climatic variation for all chelonian species, much less for all organisms. However, median nesting date has not shifted temporally as did onset of the nesting season for the northern Illinois *Chrysemys* population, a pattern resulting from increased production of subsequent nests within the same year (Schwanz & Janzen, 2008). Although this outcome may increase offspring recruitment in the short term, demographic costs may be incurred in the form of biased cohort sex ratios and a decline in the condition of adult females (Tucker et al., 2008).

Broadening the taxonomic scope, many aquatic amphibians have a thermally sensitive life cycle similar to freshwater turtles (Feder & Burggren, 1992), allowing instructive comparison concerning thermal effects on phenology. Where temporal climate shifts are substantive, amphibian phenological patterns are among those changing most swiftly (Parmesan, 2007; Todd, Scott, Pechmann, & Gibbons, 2011). Phenological rates of change for freshwater turtles were typically rapid as well, ranging from 4.7 to 9.0 days per decade for populations that exhibited significant temporal trends (Tables S2 and S4). It is further notable that, of the phenological changes recorded by Todd et al. (2011), none involved spring‐breeding amphibians at their South Carolina site, which is the same locality we also found negligible changes in nesting season onset for the three turtle taxa we monitored there. This result highlights the likely thermal concordance in spring activity of syntopic aquatic amphibians and reptiles.

### 4.1 Implications for the persistence of freshwater turtles

The preponderance of species in our study possesses an intriguing life cycle that involves offspring overwintering in the natal nest after hatching (Costanzo, Lee, & Ultsch, 2008; Gibbons, 2013). This substantially delayed emergence from the nest may be adaptive (Spencer & Janzen, 2014), yet also may incur direct metabolic costs via warmer winters (Converse, Iverson, & Savidge, 2005; Willette, Tucker, & Janzen, 2005) and thus may be affected indirectly by changing phenology. If earlier emergence of adults from hibernation is followed by earlier onset of the nesting season as implied by our findings, embryonic development during summer should also be accelerated. If embryos do not succumb directly to lethal incubation temperatures (Telemeco, Abbott, & Janzen, 2013) or suffer elevated levels of physical abnormalities (Telemeco, Warner, Reida, & Janzen, 2013), their earlier hatching could be deleterious energetically if they are obligated to stay in the nest until the following spring without feeding (Muir, Dishong, Costanzo, & Lee, 2012). As such, this notable life‐history trait of within‐nest overwintering should experience strong negative selection across many parts of the range as a consequence of increasingly earlier onset of the nesting season.

Most turtles, including all the species for which nesting phenology was examined here, have temperature‐dependent sex determination (TSD; Janzen & Paukstis, 1991; Refsnider & Janzen, 2016). Field data repeatedly document that offspring sex ratios in turtles with TSD are strongly linked to variation in climatic conditions (Janzen, 1994; but see Wyneken & Lolavar, 2015) and that such demographic effects ramify into the adult population structure on a predictable, lagged timescale (Schwanz, Spencer, Bowden, & Janzen, 2010). Shifts in initiation of the nesting season could conceivably augment populations by increasing clutch frequency, thus enhancing annual reproductive output (Tucker et al., 2008). However, models of such scenarios under realistic conditions suggest that earlier nesting seasons alone will not counteract impacts of climate change on developing reptile embryos (Telemeco, Abbott et al., 2013). Moreover, assuming nonmarine taxa no longer have the capacity to migrate to suitable habitats without anthropogenic assistance, computer simulations imply that populations with TSD almost invariably go extinct via biased sex ratios if they respond to even a modest 2°C increase in climatic temperature by employing only plasticity in nesting behaviors rather than by genetic adaptation (Morjan & Janzen, 2003). Based on these two theoretical exercises, plasticity in nesting behavior of *Chrysemys* from climatically diverse localities exhibited under common‐garden conditions (Refsnider & Janzen, 2012) may not bode well for those populations in the coming decades, in contrast with among‐population variation in TSD in *Chelydra* (Ewert, Lang, & Nelson, 2005) that may reflect local adaptation. In contrast, others suggest that turtles with TSD apparently have satisfactorily navigated prior climatic disruptions without inordinate extinction (Silber, Geisler, & Bolortsetseg, 2011) and might even benefit from female‐biased sex ratios (Hays, Mazaris, Schofield, & Laloë, 2017). However, evidence for an abrupt thermal change at the K‐Pg boundary is lacking and skewed sex ratios induce deleterious genetic effects by reducing the effective population size (Mitchell & Janzen, 2010). Regardless, turtles are already among the most globally endangered major taxa (Turtle Taxonomy Working Group, 2017), thus our findings have important conservation implications given the strong thermal dependence of the key phenological traits we examined. We predict significant future alteration of North American turtle behavior and subsequent impacts on population biology that will challenge the persistence of these increasingly imperiled organisms.

## CONFLICT OF INTEREST

None declared.

## DATA ACCESSIBILITY

All processed data are made available from the Dryad Digital Repository: https://doi.org/10.5061/dryad.kj5t8j8.
