# Supplementary material for: Altered spring phenology of North American freshwater turtles and the importance of representative populations
Source: Ecol Evol. 2018 May 4;8(11):5815–27. doi: 10.1002/ece3.4120 (PMC6010881; doi:10.1002/ece3.4120)
Supplement: Supplementary file 1 [file ECE3-8-5815-s001.docx]

Table S1. List of 21 longitudinal datasets on reptile phenology from 18 publications, defined here as having a minimum of 7 records over a 10 yr period.

| common name | species | trait(s) | shifting?^1^ | Years studied(N) | reference |
| --- | --- | --- | --- | --- | --- |
| Chinese alligator | *Alligator sinensis* | oviposition date | Y | 1987-2005(19) | Zhang et al. 2009 |
| three-lined skink | *Bassiana duperryi* | oviposition date | Y | 1997-2006(8) | Telemeco et al. 2009 |
| loggerhead sea turtle | *Caretta caretta* | median nesting date | Y | 1989-2003(15) | Weishampel et al. 2004 |
| loggerhead sea turtle | *Caretta caretta* | median nesting date | Y | 1989-2003(15) | Pike et al. 2006 |
| loggerhead sea turtle | *Caretta caretta* | first nesting date | N | 1980-2005(25) | Hawkes et al. 2007 |
| loggerhead sea turtle | *Caretta caretta* | first and last emergence date | Y^2^ | 1984-2002(19) | Mazaris et al. 2008 |
| loggerhead sea turtle | *Caretta caretta* | length of nesting season | N | 1989-2008(20) | Weishampel et al. 2010 |
| green turtle | *Chelonia mydas* | first nesting date | N | 1989-2003(15) | Pike 2009 |
| green turtle | *Chelonia mydas* | length of nesting season | N | 1989-2008(20) | Weishampel et al. 2010 |
| green turtle | *Chelonia mydas* | nesting or crawl date | N | 1984-2010(27) | Dalleau et al. 2012 |
| snapping turtle | *Chelydra serpentina* | hibernation entry and exit date | N | 1988-2007(19) | Strain et al. 2012 |
| painted turtle | *Chrysemys picta* | median nesting date | N | 1995-2006(14) | Schwanz and Janzen 2008 |
| leatherback turtle | *Dermochelys coriacea* | median nesting date | N | 1993-2013(20) | Robinson et al. 2014 |
| leatherback turtle | *Dermochelys coriacea* | median nesting date | Y | 1982-2010(29) | Robinson et al. 2014 |
| desert tortoise | *Gopherus agassizii* | gravidity and oviposition | N | 1997-2011(7) | Lovich et al. 2012 |
| Montpellier snake | *Malpolon monspessulanus* | activity period | Y | 1983-2004(16) | Moreno-Rueda et al. 2009 |
| pine snake | *Pituophis melanoleucus* | oviposition date | N | 1977-1989(13) | Burger and Zappalorti 1992 |
| sleepy lizard | *Tiliqua rugosa* | mate pairing date | Y | 1983-1997(15) | Bull and Burzacott 2002 |
| red-eared slider | *Trachemys scripta* | first nesting date | Y | 1994-2006(13) | Tucker et al. 2008 |
| red-eared slider | *Trachemys scripta* | length of nesting season | Y | 1995-2006(12) | Tucker et al. 2008 |
| asp viper | *Vipera aspis* | onset of above-ground activity | Y | 1987-2011(25) | Rugiero et al. 2013 |

1 Direction of the shift depends on the trait measured, but if present generally represents extended animal activity in response to extended warm seasons.

2 Patel et al. (2016) added 7 years of first emergence data consistent with phenological advancement.

Figure S1. Map illustrating the geographic distribution of populations studied across North America.

Figure S1. The location and species composition of the six North American field sites used to analyze temporal and climatic trends in freshwater turtle nestin­­­g phenology. The locations and species of additional phenological traits reported in this study are listed in Table 1.

## Table S2. Changes in nesting phenology estimated from linear regressions of first nesting date on year. Rate of change reflects an estimate from the regression slope. “All populations” represents a regression using data from all 14 populations of freshwater turtles. As indicated, this estimate is provided for illustrative purposes despite a lack of homogeneity of slopes (ANCOVA: Population x Year, *P*=0.01). Similarly, we provide estimates of phenological change at the species level regardless of ANCOVA results and indicate the lack of homogeneity of slopes among populations using the same notation. For species with multiple populations, separate intercepts were fit to each population significantly improving model fit. Separate regressions were also used to independently estimate change in nesting date for each population. Bold text indicates significance at the α = 0.05 level.

| species - site | rate of change (days per decade) | SE | N | *F* | *P*^3^ | *R^2^* |
| --- | --- | --- | --- | --- | --- | --- |
| All populations^1^  *Chelydra serpentina*^1^  - Algonquin Provincial Park, ON  - Crescent Lake National Wildlife Refuge, NE  - Thomson Causeway Recreation Area, IL  - Savannah River Site, SC  *Chrysemys picta*^1^  - Algonquin Provincial Park, ON  - Crescent Lake National Wildlife Refuge, NE  - Thomson Causeway Recreation Area, IL  - Two Rivers National Wildlife Refuge, IL  *Trachemys scripta*^4^  - Two Rivers National Wildlife Refuge, IL  - Savannah River Site, SC  *Kinosternon* spp.  - Crescent Lake National Wildlife Refuge, NE  - Savannah River Site, SC  *Sternotherus odoratus*  - Two Rivers National Wildlife Refuge, IL  *Malaclemys terrapin*  -Poplar Island, MD^2^ | -1.5  -1.5  -0.4  -0.6  -6.0  -4.9  -1.6  -2.8  4.6  -4.7  -6.1  -2.7  -9.0  1.7  -0.8  -1.4  0.8  -7.4  -0.3 | 0.6  0.7  1.0  1.2  1.7  4.1  1.2  1.7  2.4  1.6  5.2  2.8  3.5  4.0  1.8  1.3  5.2  5.4  1.3 | 280  91  36  23  23  9  86  26  20  25  15  35  19  16  27  17  10  13  28 | 49.6  45.0  0.14  0.28  12.9  1.44  11.2  2.88  3.57  8.97  1.36  8.91  6.53  0.19  61.4  1.24  0.03  1.87  0.06 | **0.005**  **0.025**  0.357  0.300  **<0.001**  0.135  0.087  0.051  0.960  **0.003**  0.132  0.171  **0.010**  0.667  0.334  0.142  0.562  0.099  0.401 | 0.71  0.66  0  0  0.35  0.05  0.32  0.07  0.12  0.25  0.03  0.32  0.24  0  0.82  0.01  0  0.07  0 |

^1^ Common slope estimates provided for illustrative purposes despite a significant Population x Year interaction (*P*<0.05). .

^2^ This includes data from Patuxent, MD and Poplar Island, MD.

^3^ Significance calculated from a one-tailed t-test for a negative slope.

^4^ Marginal lack of homogeneity of slopes (Population x Year, *P=0.054*)

## Table S3. Temporal trends in climate change estimated from linear regressions of heating degree-days (HDD) for February or April on year. “All sites” (above dashed line) represents a common regression using data from all 6 field sites with available nesting phenology data. Fitting a common slope was justified by a comparison of slopes test (ANCOVA: Site x Year, *P*=0.55). Rate of change reflects an estimate from the regression slope. Separate regressions were used to independently estimate change in phenology for each site. Bold text indicates significance at the α = 0.05 level.

| site | years | rate of change^4^ | SE | N | *F* | *P*^5^ | *R^2^* |
| --- | --- | --- | --- | --- | --- | --- | --- |
| All sites^1^ | 1976-2013 | -16.2 | 7.24 | 167 | 215.4 | **0.013** | 0.89 |
| Algonquin Provincial Park, ON | 1976-2011 | -8.20 | 14.7 | 36 | 0.31 | 0.29 | 0 |
| Crescent Lake National Wildlife Refuge, NE | 1981-2013 | -21.6 | 18.7 | 33 | 1.34 | 0.128 | 0.01 |
| Thomson Causeway Recreation Area, IL  Two Rivers National Wildlife Refuge, IL | 1989-2013  1994-2012 | -32.0  -67.5 | 2.20  33.5 | 25  19 | 2.21  4.06 | 0.076  **0.030** | 0.05  0.15 |
| Poplar Island, MD^2^ | 1987-2013 | -55.1 | 12.6 | 27 | 0.19 | 0.333 | 0 |
| Savannah River Site, SC | 1977-2003 | -58.1 | 12.2 | 27 | 0.23 | 0.319 | 0 |
| Sand Run Lake, WV^3^ | 1988-2007 | 10.2 | 32.4 | 20 | 0.10 | 0.622 | 0 |
| Warner, NH^3^ | 1986-2012 | -24.2 | 31.2 | 25 | 0.60 | 0.224 | 0 |
| Monkton, VT^3^ | 1986-2012 | -23.3 | 32.8 | 27 | 0.51 | 0.242 | 0 |

^1^ Site was included as an independent variable, significantly improving the model fit.

^2^ This assessment includes data from Patuxent, MD and Poplar Island, MD.

^3^ HDD for February was used because it was thought to be more relevant to the phenology studied in these populations (first emergence and first basking).

^4^ heating degree-days (HDD) per decade

^5^ Significance calculated from a one-tailed t-test for a negative slope.

## Table S4. Changes in phenology estimated from linear regressions of phenological trait on year. “All three species” represents a common regression, justified from a comparison of slopes test, using data from all three populations for which we have estimates of first emergence or first basking date (black line, Fig. 4). Rate of change reflects an estimate from the regression slope. Separate regressions were used to independently estimate change in phenology for each population and trait. Bold text indicates significance at the α = 0.05 level.

| species (site) | trait(s) | rate of change (days per decade) | SE | N | *F* | | *P*^2^ | *R^2^* |  |
| --- | --- | --- | --- | --- | --- | --- | --- | --- | --- |
| All three species^1^ | | emergence and basking | -4.6 | 2.1 | 62 | 7.33 | **0.015** | | 0.24 |
| *Chelydra serpentina* (WV) | | first emergence | -9.5 | 6.1 | 18 | 2.42 | 0.070 | | 0.08 |
| *Clemmys guttata* (NH) | | first emergence | 0.1 | 2.3 | 25 | 0.00 | 0.514 | | 0 |
| *Glyptemys insculpta* (VT) | | first basking | -7.6 | 3.3 | 19 | 5.31 | **0.017** | | 0.19 |
| *Chelydra serpentina* (WV) | | first hibernation | -2.2 | 8.1 | 19 | 0.08 | 0.392 | | 0 |

^1^ Species (i.e., population) was included as an independent variable, significantly improving the model fit.

^2^ Significance calculated from a one-tailed t-test for a negative slope.

## Table S5. Estimates of the phenological response to climatic variation from linear regressions of phenological traits on heating degree-days (HDD). “All emergence and basking” represents a common regression, justified from a comparison of slopes test, using data from all three populations for which we have estimates of first emergence or first basking date. Rate of change reflects an estimate from the regression slope. Bold text indicates significance at α = 0.05 level.

| species (site); trait | climatic factor | rate of change (days per 100 degree days) | SE | N | *F* | | *P*^1^ | *R^2^* |  |
| --- | --- | --- | --- | --- | --- | --- | --- | --- | --- |
| All emergence and basking | | HDD February | 4.8 | 1.2 | 62 | 12.2 | **<0.001** | | 0.36 |
| *Chelydra serpentina* (WV) | |  |  |  |  |  |  | |  |
| - first emergence | | HDD February | 0.5 | 4.2 | 18 | 0.01 | 0.546 | | 0 |
| - first hibernation | | HDD August | -4.3 | 10 | 19 | 0.17 | 0.341^2^ | | 0 |
| *Clemmys guttata* (NH) | |  |  |  |  |  |  | |  |
| - first emergence | | HDD February | 3.7 | 1.3 | 25 | 7.62 | **0.006** | | 0.22 |
| *Glyptemys insculpta* (VT) | |  |  |  |  |  |  | |  |
| - first basking | | HDD February | 7.4 | 1.3 | 19 | 30.8 | **<0.001** | | 0.62 |

^1^ Significance calculated from a one-tailed t-test for a positive slope in order to evaluate the *a priori* hypothesis that spring phenologies are expressed earlier when spring is warmer, except where noted.

^2^ Significance calculated from a one-tailed t-test for a negative slope in order to evaluate the *a priori* hypothesis that hibernation behavior is expressed later when the preceding month is warmer (i.e. fewer heating degree-days).

## Table S6. ANCOVA results from representative linear models of first nesting date using heating degree-days and various climate indices as covariates. Additive models that included population were always supported by Akaike weights. Conversely, estimates of the effects of various climate indices are reported here despite the fact that their inclusion did not improve model fit.

| factor | | numDf | denDf | *F* | *P* | *R^2^* |
| --- | --- | --- | --- | --- | --- | --- |
| population | 13 | | 265 | 62.8 | **<0.001** | 0.76 |
| HDD.Apr | 1 | | 265 | 57.3 | **<0.001** |  |
| NAO.DJFM^1^ | 1 | | 265 | 1.57 | 0.211 |  |
| population | 13 | | 265 | 63.1 | **<0.001** | 0.76 |
| HDD.Apr | 1 | | 265 | 57.6 | **<0.001** |  |
| ONI.FMA^2^ | 1 | | 265 | 2.71 | 0.101 |  |
| population | 13 | | 265 | 62.7 | **<0.001** | 0.75 |
| HDD.Apr | 1 | | 265 | 57.2 | **<0.001** |  |
| cENSO.FMA^3^ | 1 | | 265 | 1.04 | 0.309 |  |
| population | 13 | | 265 | 62.9 | **<0.001** | 0.76 |
| HDD.Apr | 1 | | 265 | 57.4 | **<0.001** |  |
| SOI.Apr^4^ | 1 | | 265 | 1.83 | 0.178 |  |
| population | 13 | | 265 | 63.1 | **<0.001** | 0.76 |
| HDD.Apr | 1 | | 265 | 57.5 | **<0.001** |  |
| PNA.Apr^5^ | 1 | | 265 | 2.61 | 0.108 |  |

^1^ seasonal North Atlantic Oscillation index for December, January, February, and March (DJFM)

^2^ seasonal Oceanic Niño index for February, March, and April (FMA)

^3^ seasonal El Niño/Southern Oscillation index for February, March, and April (FMA)

^4^ monthly mean Southern Oscillation Index for April

^5^ monthly mean Pacific/North American Oscillation index for April

## Table S7. Linear regression estimate of the relationship between observed first nest date and observed first gravid date of terrapins from Patuxent, MD between 1987 and 1994.

| factor | | slope | | SE | | numDf | denDf | *F* | *P* | *R^2^* |
| --- | --- | --- | --- | --- | --- | --- | --- | --- | --- | --- |
| first gravid date | 0.86 | | 0.4 | | 1 | | 6 | 4.53 | 0.08 | 0.43 |

## Table S8. Estimates of the phenological response to climatic variation from linear regressions of first nesting date on the monthly mean for April. Rate of change reflects an estimate from the regression slope. “All populations” represents a regression using data from all 14 populations. Separate regressions were used to independently estimate change in nesting date for each species and population. Bold text indicates significance at α = 0.05 level.

| species - site | rate of change (days per degree C) | SE | N | *F* | *P*^3^ | *R^2^* |  |
| --- | --- | --- | --- | --- | --- | --- | --- |
| All populations^1^  *Chelydra serpentina*^1^  - Algonquin Provincial Park, ON  - Crescent Lake National Wildlife Refuge, NE  - Thomson Causeway Recreation Area, IL  - Savannah River Site, SC  *Chrysemys picta*^1^  - Algonquin Provincial Park, ON  - Crescent Lake National Wildlife Refuge, NE  - Thomson Causeway Recreation Area, IL  - Two Rivers National Wildlife Refuge, IL  *Trachemys scripta*^1^  - Two Rivers National Wildlife Refuge, IL  - Savannah River Site, SC  *Kinosternon* spp.^1^  - Crescent Lake National Wildlife Refuge, NE  - Savannah River Site, SC  *Sternotherus odoratus*  - Two Rivers National Wildlife Refuge, IL  *Malaclemys terrapin*  -Poplar Island, MD^2^ | | -2.0  -1.9  -1.7  -1.7  -2.5  -1.7  -1.9  -2.3  -0.79  -2.1  -2.6  -2.4  -3.4  0.1  -1.8  -1.1  -5.1  -1.9  -2.6 | 0.3  0.3  0.6  0.4  0.7  2.3  0.5  0.7  1.1  0.7  1.2  1.1  1.0  2.4  0.8  0.5  2.6  1.6  0.8 | 280  91  36  23  23  9  86  26  20  25  15  35  19  16  27  17  10  13  28 | 45.4  64.4  8.83  15.5  11.8  0.08  17.1  10.8  0.52  7.85  4.72  11.8  10.9  0  75.7  4.09  3.72  1.58  10.9 | **<0.001**  **<0.001**  **0.002**  **<0.001**  **0.001**  0.235  **<0.001**  **0.001**  0.239  **0.005**  **0.024**  **0.019**  **0.002**  0.511  **0.018**  **0.031**  **0.045**  0.118  **0.001** | 0.76  0.74  0.18  0.39  0.33  0  0.43  0.28  0  0.22  0.21  0.39  0.36  0  0.85  0.16  0.23  0.05  0.27 |

^1^ Population was included as an independent variable in these models, significantly improving the statistical fit.

^2^ This includes data from Patuxent, MD and Poplar Island, MD.

^3^ Significance calculated from a one-tailed t-test for a positive slope.

Figure S2. Linear regression of the rate of change in the onset of nesting date (i.e. the slope of the temporal relationship between the onset of nesting and year) as as a function of (a) latitude and (b) the rate of change in HDD for April experience at these field sites.


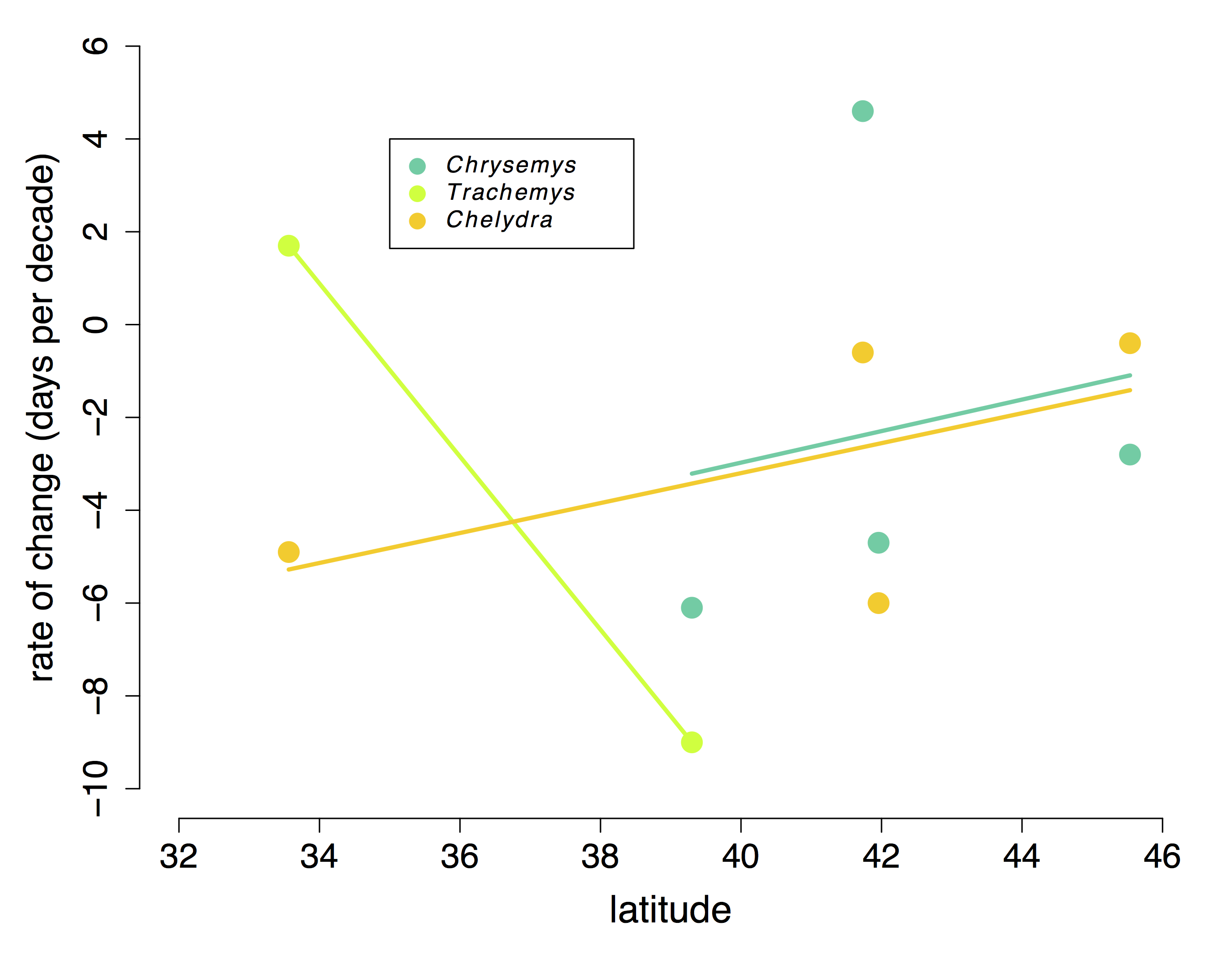

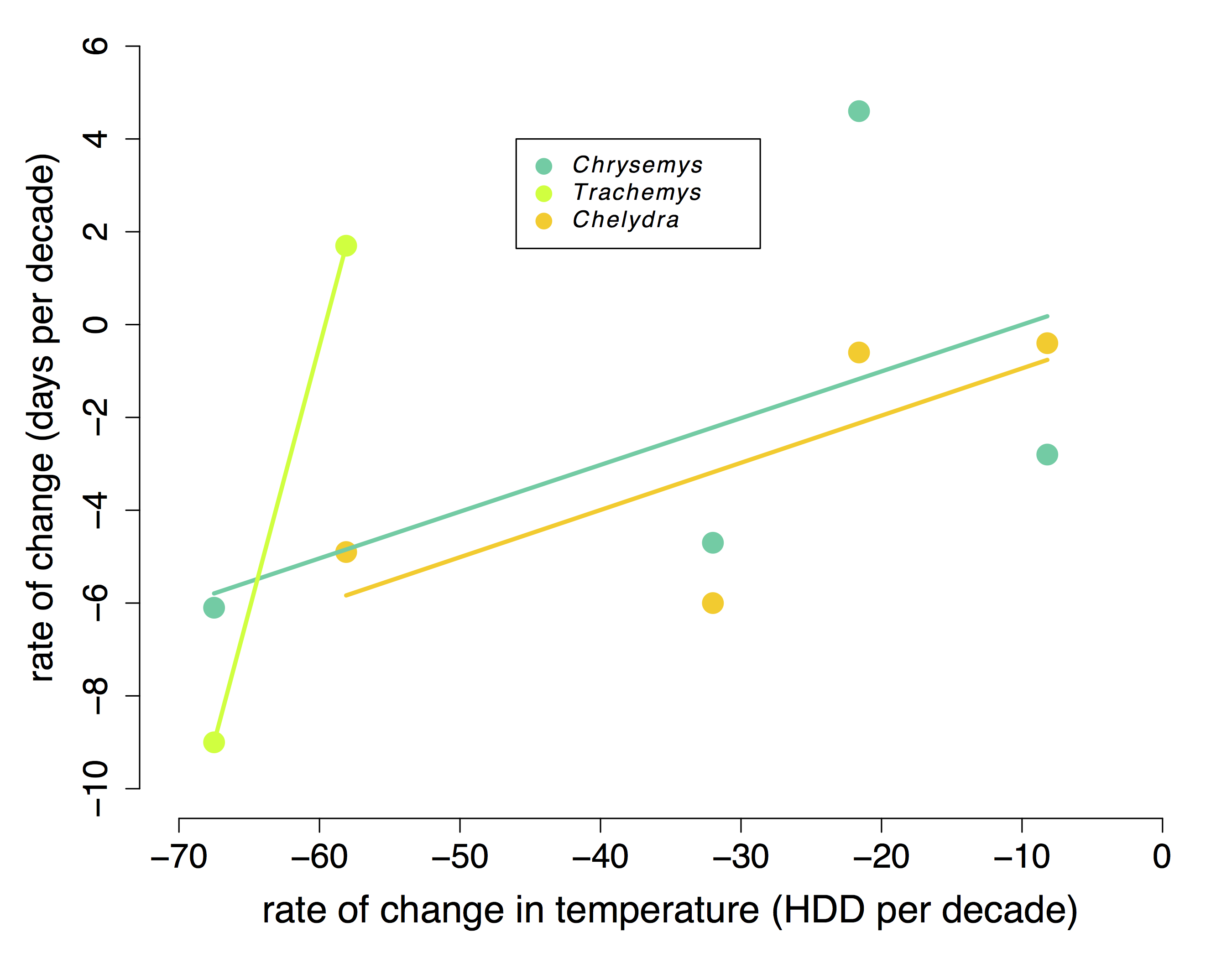


Supplementary References

Bull, C. M., and D. Burzacott. 2002. Changes in climate and in the timing of pairing of the Australian lizard, *Tiliqua rugosa*: a 15-year study. Journal of Zoology **256**:383-387.

Burger, J., and R. T. Zappalorti. 1992. Philopatry and nesting phenology of pine snakes *Pituophis melanoleucus* in the New Jersey Pine Barrens. Behavioral Ecology and Sociobiology **30**:331-336.

Dalleau, M., S. Ciccione, J. A. Mortimer, J. Garnier, S. Benhamou, and J. Bourjea. 2012. Nesting phenology of marine turtles: insights from a regional comparative analysis on green turtle (*Chelonia mydas*). PLoS ONE **7**:e46920.

Hawkes, L. A., A. C. Broderick, M. H. Godfrey, and B. J. Godley. 2007. Investigating the potential impacts of climate change on a marine turtle population. Global Change Biology **13**:923-932.

Lovich, J., M. Agha, M. Meulblok, K. Meyer, J. Ennen, C. Loughran, S. Madrak, and C. Bjurlin. 2012. Climatic variation affects clutch phenology in Agassiz’s desert tortoise *Gopherus agassizii*. Endangered Species Research **19**:63-74.

Mazaris, A. D., A. S. Kallimanis, S. P. Sgardelis, and J. D. Pantis. 2008. Do long-term changes in sea surface temperature at the breeding areas affect the breeding dates and reproduction performance of Mediterranean loggerhead turtles? Implications for climate change. Journal of Experimental Marine Biology and Ecology **367**:219-226.

Moreno-Rueda, G., J. Pleguezuelos, and E. Alaminos. 2009. Climate warming and activity period extension in the Mediterranean snake *Malpolon monspessulanus*. Climatic Change **92**:235-242.

Pike, D. A. 2009. Do green turtles modify their nesting seasons in response to environmental temperatures? Chelonian Conservation and Biology **8**:43-47.

Pike, D. A., R. L. Antworth, and J. C. Stiner. 2006. Earlier nesting contributes to shorter nesting seasons for the loggerhead seaturtle, *Caretta caretta*. Journal of Herpetology **40**:91-94.

Robinson, N. J., S. E. Valentine, P. S. Tomillo, V. S. Saba, J. R. Spotila, and F. V. Paladino. 2014. Multidecadal trends in the nesting phenology of Pacific and Atlantic leatherback turtles are associated with population demography. Endangered Species Research **24**:197-206.

Rugiero, L., G. Milana, F. Petrozzi, M. Capula, and L. Luiselli. 2013. Climate-change-related shifts in annual phenology of a temperate snake during the last 20 years. Acta Oecologica **51**:42-48.

Schwanz, L. E., and F. J. Janzen. 2008. Climate change and temperature-dependent sex determination: Can individual plasticity in nesting phenology prevent extreme sex ratios? Physiological and Biochemical Zoology **81**:826-834.

Strain, G. F., J. T. Anderson, E. D. Michael, and P. J. Turk. 2012. Hibernacula use and hibernation phenology in the common snapping turtle (*Chelydra serpentina*) in Canaan Valley, West Virginia. Journal of Herpetology **46**:269-274.

Telemeco, R. S., M. J. Elphick, and R. Shine. 2009. Nesting lizards (*Bassiana duperreyi*) compensate partly, but not completely, for climate change. Ecology **90**:17-22.

Tucker, J. K., C. R. Dolan, J. T. Lamer, and E. A. Dustman. 2008. Climatic warming, sex ratios, and red-eared sliders (*Trachemys scripta elegans*) in Illinois. Chelonian Conservation and Biology **7**:60-69.

Weishampel, J. F., D. A. Bagley, and L. M. Ehrhart. 2004. Earlier nesting by loggerhead sea turtles following sea surface warming. Global Change Biology **10**:1424-1427.

Weishampel, J. F., D. A. Bagley, L. M. Ehrhart, and A. C. Weishampel. 2010. Nesting phenologies of two sympatric sea turtle species related to sea surface temperatures. Endangered Species Research **12**:41-47.

Zhang, F., Y. Li, Z. Guo, and B. R. Murray. 2009. Climate warming and reproduction in Chinese alligators. Animal Conservation **12**:128-137.
